# Dental Pulp Stem Cells on Implant Surface: An In Vitro Study

**DOI:** 10.1155/2021/3582342

**Published:** 2021-03-23

**Authors:** Luigi Laino, Marcella La Noce, Luca Fiorillo, Gabriele Cervino, Ludovica Nucci, Diana Russo, Alan Scott Herford, Salvatore Crimi, Alberto Bianchi, Antonio Biondi, Gregorio Laino, Antonino Germanà, Marco Cicciù

**Affiliations:** ^1^Multidisciplinary Departments of Medical-Surgical and Dental Specialties, Second University of Naples, Naples 80100, Italy; ^2^Department of Biomedical and Dental Sciences and Morphological and Functional Imaging, Messina University, Messina 98100, Italy; ^3^Department of Maxillofacial Surgery, Loma Linda University, Loma Linda, CA 92354, USA; ^4^Department of Surgical and Biomedical Sciences, Catania University, 95124 Catania, Italy; ^5^Department of Veterinary Sciences, University of Messina, Messina, Italy

## Abstract

In the field of biology and medicine, one hears often about stem cells and their potential. The dental implant new surfaces, subjected to specific treatments, perform better and allow for quicker healing times and better clinical performance. The purpose of this study is to evaluate from a biological point of view the interaction and cytotoxicity between stem cells derived from dental pulp (DPSCs) and titanium surfaces. Through the creation of complex cells/implant, this study is aimed at analyzing the cytotoxicity of dental implant surfaces (Myth (Maipek Manufacturer Industrial Care, Naples, Italy)) and the adhesion capacity of cells on them and at considering the essential factors for implant healing such as osteoinduction and vasculogenesis. These parameters are pointed out through histology (3D cell culture), immunofluorescence, proliferation assays, scanning electron microscopy, and PCR investigations. The results of the dental implant surface and its interaction with the DPSCs are encouraging, obtaining results increasing the mineralization of the tissues. The knowledge of this type of interaction, highlighting its chemical and biological features, is certainly also an excellent starting point for the development of even more performing surfaces for having better healing in the oral surgical procedures related to dental implant positioning.

## 1. Introduction

### 1.1. Background

Stem cells are found in various body tissues: blood, muscles, skin, bone marrow, nerves, and liver. The key property of all stem cells is that they are undifferentiated; therefore, they can replicate indefinitely and replace/renew different types of damaged cells in the body [1–4]. Stem cells can divide and replicate over 200 types of specialized cells that are linked to the function of the immune system, heart, oxygen distribution, and others. Literature shows that stem cells from the dental pulp share behavioral characteristics similar to mesenchymal stem cells (MSCs) from other tissues [5–8]. MSCs are present in many tissues throughout the organism and can transform and replicate muscle, nerve, bone, and fat and cartilage cells. They also have the ability to modify the behavior of the immune system and thus potentially treat a range of immune disorders [9]. Stem cells in the teeth could, in the future, be used to repair damage throughout the body and be used in regenerative medicine. The dental pulp is a connective tissue, contained within the pulp chamber and in root canals; it communicates with the periodontium through one or more apical foramina and through the lateral accessory channels of the roots [10, 11]. The pulp is composed of cells immersed in an intercellular matrix characterized by a fundamental substance and fibers (especially collagen fiber types I and III) [12]. The organic matrix represents about 25%, while the remaining 75% is made up of water. The central mass of the pulp is made up of cells and an intercellular matrix. The dental pulp plays the main role in tooth regeneration after an insult by participating in the process known as dentinogenesis [13–15]. The direct capping of the pulp with Mineral Trioxide Aggregate (MTA) or calcium hydroxide, which promotes the activation of dentinogenesis with the production of tertiary dentin, is promoted by these tissues. This newly mineralized layer preserves pulp integrity and serves as a barrier to insult [16–19].

Inside the healthy pulp, there are fibroblasts, fibrocytes, mesenchymal stem cells, lymphocytes, macrophages-histiocytes, and rare mast cells. The intercellular matrix, which surrounds and supports the structures, is composed of collagen fibers, type I and to a lesser extent type III, and a fundamental substance, made up of water and proteoglycans. The fundamental substance represents the means by which metabolites and waste products are spread in the pulp [5–7]. With advancing age, there is a progressive decrease in the cell population and a numerical and volumetric increase in collagen fibers, especially in the 2/3 apical roots. Two different types of stem cells are distinguished: embryonic stem cells (ESCs) and adult stem cells (ASCs) [20].

ESCs are obtained directly from human embryos. Up to 3-4 days after fertilization (zygote and blastomeres of the morula), stem cells are totipotent: they have morphogenetic capacity. They are capable of giving rise to a complete individual, they have unlimited multiplicative and proliferative capacity (cell immortality), and they can differentiate into all cell types (differentiating ability) [21, 22]. At the implant surgery level, autologous bone derived from stem cells could replace the current materials used for guided bone regeneration (GBR) [23–26]. In addition, the possibility of having ligament-anchored implants, or implants surrounded by periodontal tissue, produced thanks to tissue engineering, between bone and implant surface, seems to arouse the interest of many researchers [27–29]. The characteristics of the implant surfaces have different implications in the integration that it will be possible to achieve, during rehabilitation, with both hard and soft tissues [7, 30]. A rough implant allows for greater osseointegration rates than a smooth surface one. Equally important is the management of soft tissues and the transmucosal portion of the implant [31, 32].

Over time, the study in the dental implant field has led to a change from smooth machined surfaces to roughened surfaces in order to improve osseointegration thanks to the osteoconductive properties of this type of texture [33, 34]. Scarano et al. recently demonstrated how a faster osseointegration could be achieved in the presence of specifically treated implant surfaces, promising encouraging clinical outcomes [35]. Other related researches highlighted how the presence of stem cells applied to a dental implant surface could increase and accelerate the physiological osteointegration processes [36, 37]. Scarano et al. [35] showed how the addition of bone marrow stromal stem cells could improve bone regeneration during bone porcine block regeneration techniques. Another study suggest that thermal treatment of dental implant surface could provide a better osseointegration [36]. The study evaluated the influence of this treatment of Ti6Al4V implant surfaces and the bone healing response in a rabbit model. They highlighted a statistically significant difference of bone-implant contact (BIC).

Other recent studies suggest that inflamed peri-implant tissues with associated progressive bone loss are becoming an increasingly frequent situation. One of the possible explanations for the phenomenon seems to derive from the fact that rough implants could favor the formation and deposit of bacterial plaque, which could then start the inflammatory process in the peri-implant tissues [37, 38].

### 1.2. Hypothesis

The aim of this scientific study is to evaluate the biological and interaction characteristics between Myth (Maipek Manufacturer Industrial Care, Naples, Italy) surface and stem cells derived from dental pulp (DPSCs). The clinical rationale of the study is to underline how the presence of MSCs and its interaction with the dental implant surface may increase the inflammatory tissue response with a quicker healing on the surgical site. This study was performed to evaluate the inflammatory response to novel dental implant surface, and the authors performed 2D and 3D cell culture, immunofluorescence, proliferation assays, scanning electron microscopy (SEM), and PCR (Polymerase Chain Reaction) investigations.

## 2. Materials and Methods

**Figure 1:**
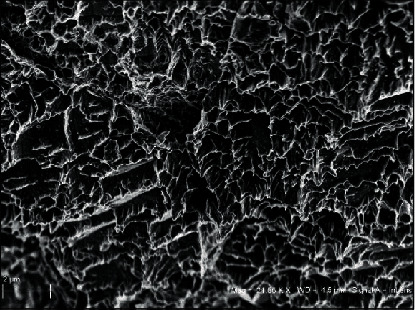
Myth (Maipek Manufacturer Industrial Care, Naples, Italy) implant texture observed by SEM.

This work presents an *in vitro* study about the ability to stimulate the osteogenesis of DPSCs by Myth (Maipek Manufacturer Industrial Care, Naples, Italy) implant texture. Surface structure was viewed by SEM (scanning electron microscopy) and is reported in Figure 1, which highlights its roughness.

To conduct this study, complex cells/implant was realized: in particular, as Myth (Maipek Manufacturer Industrial Care, Naples, Italy) is aimed at a dental use, DPSCs, a lineage of mesenchymal stem cells extracted by dental pulp, were chosen. The methods of Naddeo et al. [39] have been followed.

### 2.1. Sample

Myth® (Maipek Manufacturer Industrial Care, Naples, Italy) is made of Grade 4 titanium. Titanium has a relative density of 4.5 g/cm [3] and a very low thermal conductivity and has a very high mechanical strength with an elongation at break equal to 12%. The modulus of elasticity is relatively low and similar to that of the bone. Grade 4 titanium within the four varieties of pure titanium (Ti cp) has the best overall characteristics, combining the workability and therefore precision typical of low grades with the superior mechanical properties of high grades. The fundamental characteristics of this metal are the high corrosion resistance and the high degree of biocompatibility. An atomic bombing with inert gas and magnetic fields were used to decontaminate the devices. About 18 implants were employed in this work.

#### 2.1.1. Cell Extraction and 2D Culture

Mesenchymal stem cells were obtained by the extraction of dental pulp tissue from third molars. All subjects signed the ethical committee consent brochure (Second University Internal Ethical Committee). After mechanical and enzymatic digestion of the tissue with a collagenase I/dispase solution, the sample was filtered with 70 m Falcon strainers (BD Pharmingen, Buccinasco, Milano, Italy) and centrifuged for 7 min at 1300 rpm. The pellets were then plated in T-25 flasks at 37°C and 5% CO_2_ in DMEM culture medium supplemented with 10% fetal bovine serum (FBS), 2 mM l-glutamine, and 100 U/mL penicillin and 100 mg/mL streptomycin (all purchased from Gibco-Life Technologies, Monza, Italy). Adhered cells were expanded until they reached about 5 × 105 cells/flask.

#### 2.1.2. FACS Analysis and Sorting

Cells were detached using trypsin EDTA (GIBCO). At least 200,000 cells were incubated with fluorescent conjugated antibodies for 30 min at 4°C, washed, and resuspended in PBS. The antibodies used in this study were anti-CD34 PE (BD Pharmingen, Buccinasco, Milano, Italy) and anti-CD90 FITC (BD Pharmingen, Buccinasco, Milano, Italy). Isotypes were used as controls. Cells were analyzed with an Accuri C6 (BD Biosciences, San Jose, CA, USA) and the data collected with FCS Express version 3 (De Novo Software). Cells were sorted using simultaneous positivity for CD90 and CD34 using a FACSAria III (BD, Franklin Lakes, NJ, USA). The purity of sorted populations was routinely 90%.

#### 2.1.3. 3D Cell Culture: In Vitro Tissue Engineering

In order to achieve 3D tissue formation, cells were seeded at a density of 5 × 10^5^ cells/implant onto dental implants that had been previously washed in PBS. Cells were resuspended in 100 *μ*L of culture medium and plated as a drop on the scaffold placed in a 12-well plate, taking care not to spill the medium at the bottom of the plate, to allow cell attachment. After 1 h of incubation, the cell implant devices were transferred to 15 mL tubes with a cap filter and incubated with osteogenic medium in a humidified atmosphere at 37°C and 5% CO_2_ in a rotating culture apparatus (Wheaton Science Products, Millville, NJ, USA) at 6 rpm; cells plated in flasks were used as the control (2D culture). The 3D culture was performed for 30 days in osteogenic medium changed twice weekly; specimens were collected at 7, 14, and 30 days. Osteoinduction medium is composed of DMEM supplemented with 10% FBS, 1% Pen-Strep, 50 *μ*g/mL L-ascorbic acid (Sigma, Gillingham, Dorset, UK), 10 mM glycerol phosphate disodium salt (*β*-glycerophosphate), and 10 nM dexamethasone (Sigma, Gillingham, Dorset, UK). Experiments were performed in triplicate (*n* = 3 scaffolds/time point).

#### 2.1.4. Cytotoxicity Test on Conditioned Medium

Cytotoxicity was evaluated on cells cultured in medium conditioned by the presence of implants. The conditioned medium was prepared by incubating each implant in 3 mL of DMEM without phenol red and supplemented with antibiotics (penicillin, streptomycin), glutamine, and FBS at 37°C for 3 days. DPSCs were seeded in 96-well plates at a density of 10 [4] cells per well and cultured in conditioned medium for 24 h and 48 h, and the cell viability was determined by MTT colorimetric assay. The values are expressed as the percentage of cell viability compared with control (cells incubated in unconditioned culture medium). The measurements were performed in triplicate.

#### 2.1.5. Proliferation Assays

The MTT colorimetric assay was also performed to assess cell adhesion and proliferation. To this end, 5 × 10^5^ cells were plated on implants and incubated, as described above, in DMEM supplemented with FBS, l-glutamine, and antibiotics. Seeded implants were collected after 24 h and 48 h of 3D culture: medium was removed and cell implants incubated for 4 h in a solution of 5 mg/mL MTT. The same number of cells cultured in 2D was used as the control. After medium removal, 300 *μ*L of DMSO was added to each well containing seeded implants or control cells for 10 min; supernatants collected were read at 540 nm with a spectrophotometer. Cell viability was calculated proportionally to the quantity of formazan salts produced by the enzymatic activity of cells. Values are given as percentage versus the control and normalized with respect to the number of cells and sample volumes.

#### 2.1.6. Immunofluorescence

Expression of osteocalcin on seeded cells was evaluated at 3 and 30 days of culture. Implants seeded with 1 × 10^6^ cells/mL were washed in PBS and fixed with 4% paraformaldehyde (PFA) solution. Samples were incubated with primary antibodies: mouse monoclonal to osteocalcin (1 : 100, Abcam, Cambridge, UK), overnight at 4°C in the dark. This step was followed by incubation with the secondary antibody tetramethylrhodamine- (TRITC-) conjugate (1 : 1000, Abcam). Nuclear counterstaining was performed with 4,6-diamidino-2-phenylindole (DAPI). After extensive washing with PBS, images were collected under a fluorescence microscope (Axiovert 100; Zeiss). In order to mimic the three-dimensional bone structure as much as possible (3D culture) and to assess whether the scaffolds were capable of inducing adhesion, about 250,000 cells were plated on 2 implants and incubated in rotating culture at 37°C in 5% CO_2_. After 3 and 30 days of culture, the medium was removed and the implants were washed with Phosphate-Buffered Saline (PBS) and fixed in 4% paraformaldehyde (PFA). Then, fluorescence was performed by labeling with Hoechst, an intercalating-DNA dye that displays cell nuclei. The ability to express osteogenic specific markers was evaluated by immunofluorescence staining for osteocalcin, both at 3 and at 30 days of culture.

#### 2.1.7. Scanning Electron Microscopy

Adhered cell morphology was assessed by SEM (Supra 40 ZEISS, Weimar, Germany). Seeded implants were deprived of medium, washed, fixed in PFA, and postfixed with 0.1% OsO_4_ for 1 h. Thereafter, specimens were gradually dehydrated in an increasing ethanol concentration, treated by critical point drying, dry mounted on a stub, and sputter-coated with gold/palladium. DPSC/implant complexes, cultured for 3 and 30 days in the same conditions described above, after fixation were processed for SEM analyses, to obtain a clearer view of cell adhesion.

#### 2.1.8. qRT-PCR

The osteoinduction capability of implants was evaluated by qRT-PCR analysis for genes involved in osteogenic differentiation on specimens collected after 7, 14, and 30 days of 3D cell culture. In particular, we examined the expression of genes involved in the production of molecules responsible for deposition of mineralized matrix: bone alkaline phosphatase (BAP), collagen I (COLL I), osteopontin (OPN), bone sialoprotein (BSP), and osteocalcin (OSTC). RNA extracted from pellets of cells cultured in 2D was used as control. RNA from cells adhered on implants was extracted by processing the entire sample according to the protocol of the Ambion RNA extraction kit (Life Technologies). cDNA was obtained after treatment with DNase (Promega, Italy) and reverse transcriptase (ImProm-II Reverse Transcriptase). Samples were analyzed using real-time quantitative PCR (qRT-PCR). PCR reactions were performed using a StepOne Thermocycler (Applied Biosystems, Monza, Italy), and the amplifications were done using the SYBR Green PCR Master Mix (Applied Biosystems, Monza, Italy). The thermal cycling conditions were 50°C for 2 min followed by an initial denaturation step at 95°C for 2 min and 40 cycles at 95°C for 30 s, 60°C or 58°C for 30 s, and 72°C for 60s. Real-Time PCR was performed using the primer sequences shown in Table 1. An additional step starting from 60 to 95°C (0.05°C·s^−1^) was performed to establish a melting curve. This was used to verify the specificity of the qRT-PCR reaction for each primer pair. For each measurement, a threshold cycle value (Ct) was determined. This was defined as the number of cycles necessary to reach a point at which the fluorescent signal is first recorded as being statistically significant above the background. Data were analyzed by using the 2 − *ΔΔ*Ct method to obtain the relative expression level, and each sample was normalized by using the GAPDH RNA expression. The ability of the implant texture to induce differentiation of DPSCs into the osteoblast to activate bone matrix deposition was evaluated by Real-Time Polymerase Chain Reaction (Real-Time PCR). The analyses were conducted on specimens collected after 7, 14, and 30 days of cell culture; in particular, the expression of genes encoding for molecules involved in matrix mineralization was examined: BAP, COLL 1, OPN, BSP, and OSTC. RNA extracted from pellets of cells cultured in flasks (2D) was used as control. Quantitative Real-Time PCR was performed using the SYBR Green method. The amount of cDNA of the gene of interest has been normalized to that of the cDNA of GAPDH. The experiments were carried out in triplicate for each data point (Table 1).

#### 2.1.9. Alizarin Red S Quantification

After 30 days of 3D culture, cell-implant biocomplexes were washed with PBS and fixed in 10% (*v*/*v*) formaldehyde (Sigma-Aldrich) at room temperature for 15 min. The samples were then washed twice with excess dH_2_O prior to addition of 1 mL of 40 mM ARS (pH 4.1). Samples were incubated at room temperature for 20 min with gentle shaking. After aspiration of the unincorporated dye, the samples were washed four times with 4 mL dH_2_O while shaking for 5 min and then stored at −20°C prior to dye extraction. For quantification of staining, 800 *μ*L 10% (*v*/*v*) acetic acid was added to each sample, and the plate was incubated at room temperature for 30 min with shaking. Cells, now loosely attached to the implants, were then scraped with a cell scraper (Fisher Life Sciences) and transferred with 10% (*v*/*v*) acetic acid to a 1.5 mL microcentrifuge tube with a wide-mouth pipette. After vortexing for 30 s, the slurry was overlaid with 500 *μ*L mineral oil (Sigma-Aldrich), heated to exactly 85°C for 10 min, and transferred to ice for 5 min. The slurry was then centrifuged at 20,000 g for 15 min, and 500 *μ*L of the supernatant was removed to a new 1.5 mL microcentrifuge tube. Then, 200 *μ*L of 10% (*v*/*v*) ammonium hydroxide was added to neutralize the acid. pH was measured to ensure that it was between 4.1 and 4.5. Aliquots (150 *μ*L) of the supernatant were read in triplicate at 405 nm in 96-well format using opaque-walled, transparent-bottomed plates (Fisher Life Sciences). Cells seeded in 2D were used as control. In order to evaluate the ability to induce osteogenic differentiation, cells seeded on Myth (Maipek Manufacturer Industrial Care, Naples, Italy) surfaces were cultured for three weeks in osteogenic medium in rotating culture. After PBS washing, the complexes were fixed and kept in a solution of Alizarin Red S 1% for 10 min. Alizarin is a red staining that binds calcium deposition by cells of an osteogenic lineage. Free calcium forms precipitates with alizarin and tissue containing calcium stain red immediately, when immersed in a solution containing it [40].

#### 2.1.10. ELISA for h-OSTC and h-VEGF

In order to evaluate levels of human OSTC and VEGF produced by the cells and released into the culture medium, supernatant was collected from 3D cultures after 7, 14, and 30 days of culture. After centrifugation to remove particulates, 2 mL aliquots of medium were stored at −20°C until processing for analysis. The evaluation was carried out with an ELISA kit (Human Osteocalcin ELISA kit, Invitrogen; Human VEGF ELISA kit, Invitrogen), and concentrations were read versus a standard curve at 450 nm using a spectrophotometer (DAS Plate Reader, Rome, Italy). The assays were performed in triplicate [41, 42].

## 3. Results

### 3.1. Cytotoxicity Test: Conditioned Medium

**Figure 2:**
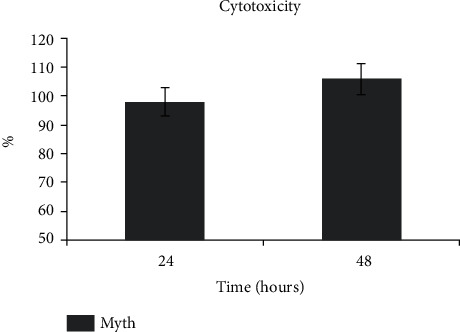
Cytotoxicity test on Myth (Maipek Manufacturer Industrial Care, Naples, Italy) implant by conditioned medium after 24 and 48 hours of incubation.

The high values of percentage showed in the graph (Figure 2) prove a total biocompatibility of the implants, suggesting that no particles that damage cells were released by them.

So, the Myth (Maipek Manufacturer Industrial Care, Naples, Italy) implant can be considered biologically safe.

### 3.2. Cell Proliferation Assay: MTT Tests

**Figure 3:**
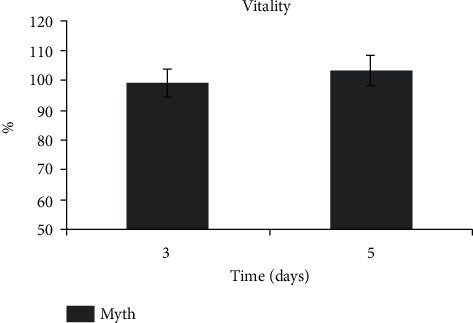
Proliferation assays on construct DPSCs/Myth (Maipek Manufacturer Industrial Care, Naples, Italy) at 3 and 5 days of culture.

The amount is expressed in percentage versus the control cultured in the plate [43]. The implants promote cell proliferation approximately with the same values of the cell culture in standard conditions (Figure 3).

### 3.3. Cell Adhesion: Immunofluorescence

**Figure 4:**
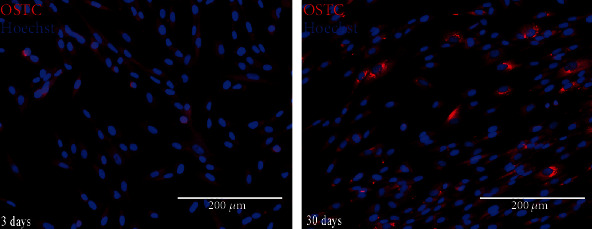
Immunofluorescence by Hoechst and OSTC on device DPSCs/Myth (Maipek Manufacturer Industrial Care, Naples, Italy) at 3 and 30 days of culture.

The images show the nuclei of adhered cells, evenly distributed on the implant's surfaces. Cells expressed osteocalcin as early as 3 days. The expression of osteocalcin is increased at 30 days, confirming the stability and osteogenic induction of the implant (Figure 4).

### 3.4. Cell Adhesion: Scanning Electron Microscopy (SEM)

**Figure 5:**
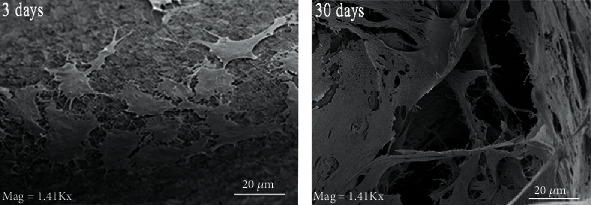
SEM photos of cells adhered on Myth (Maipek Manufacturer Industrial Care, Naples, Italy) surfaces after 3 days of culture. Cells form a monolayer after 30 days of culture.

As the collected photos showed, adhered cells tended to spread onto Myth (Maipek Manufacturer Industrial Care, Naples, Italy) surfaces acquiring an osteoblastic morphology (Figure 5) [44].

### 3.5. Bone Matrix Formation: Histological Analysis

**Figure 6:**
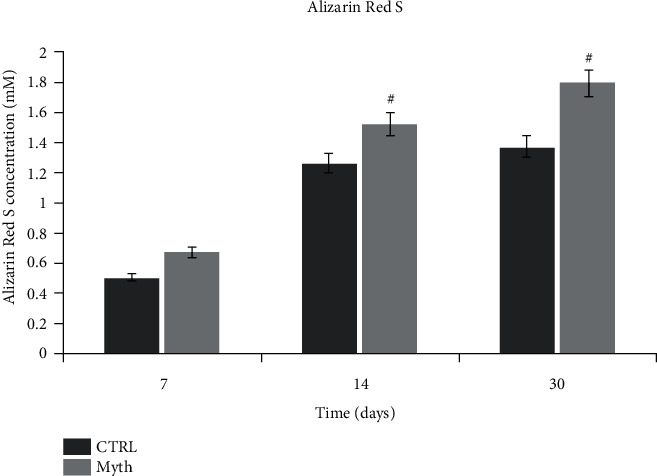
Alizarin Red S quantification.

Alizarin Red S quantification has been performed because the thickness of the implant does not allow a quality image. As shown in Figure 6, cells seeded on Myth lay a quantity of calcified matrix greater than the control in which cells were grown in adhesion in the same condition described above (2D culture).

### 3.6. Osteoinduction: qRT-PCR

**Figure 7:**
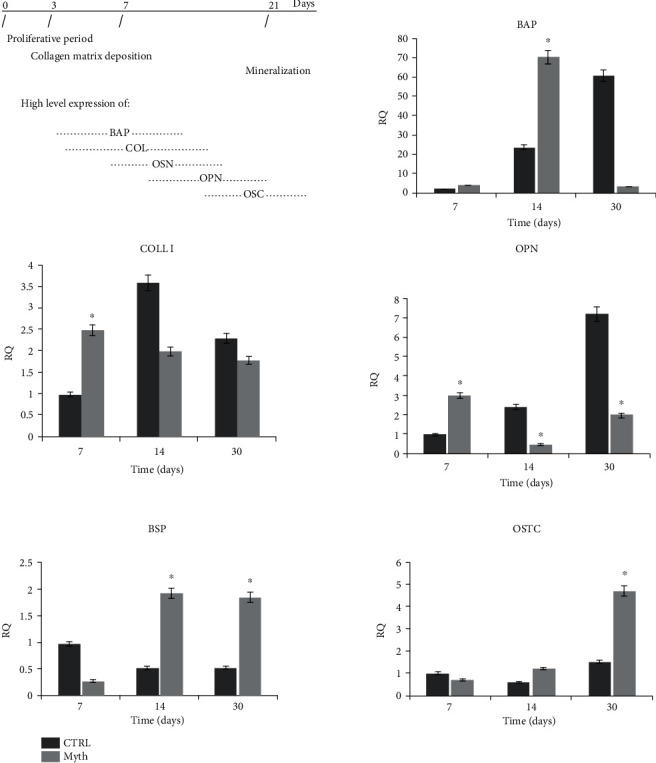
qRT-PCR of osteogenic genes BAP, COLL I, OPN, BSP, and OSTC in cells seeded on Myth (Maipek Manufacturer Industrial Care, Naples, Italy) versus a 2D control (CTRL) at 7, 14, and 30 days of culture.

The image (Figure 7) in the upper left shows the temporal expression of markers involved in osteogenic differentiation; histograms display the activation of genes *BAP* (bone alkaline phosphatase), *COLLI* (collagen), *OPN* (osteopontin), *BSP* (bone sialoprotein), and *OSTC* (osteocalcin) in cells seeded on Myth (Maipek Manufacturer Industrial Care, Naples, Italy) implants versus a control 2D at 7, 14, and 30 days of culture.

The histograms show in cell-implant devices an upregulation of genes *BSP* and *OSTC* compared to the 2D system. Moreover, for the implants, the deposition of the matrix is already carried out after 7 days of culture (*COLL I*), compared to the control that, instead, presents the highest expression of *COLL I* just after 14 days of culture, a growing trend of *OPN* and lower expression of *BSP* and *OSTC* with respect to Myth (Maipek Manufacturer Industrial Care, Naples, Italy) specimens. Then, the global analysis shows that the implant system enters early in a stage of matrix mineralization stimulating previously cell differentiation.

### 3.7. Matrix Mineralization: Human-Osteocalcin ELISA Test

Osteocalcin is the latest marker of the mature osteoblasts. It is the most abundant noncollagenous protein of the bone matrix. Once transcribed, osteocalcin undergoes posttranslational modifications within the osteoblast before its secretion. Osteocalcin is released by osteoblasts during bone formation and is bounded with the mineralized bone matrix.

**Figure 8:**
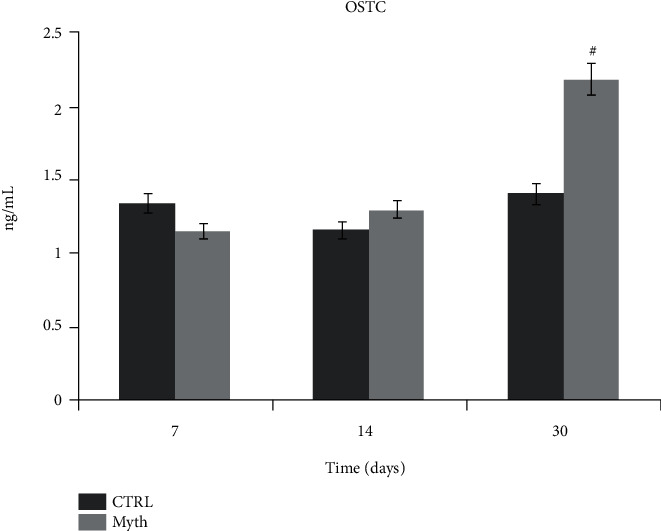
h-OSTC ELISA test of culture medium collected from 2D control (CTRL) and DPSC/Myth (Maipek Manufacturer Industrial Care, Naples, Italy) devices after 7, 14, and 30 days of cell culture. The concentration was expressed in ng/mL.

The concentration of osteocalcin released in the culture medium by cells seeded on implants (3D) was evaluated by ELISA test, after 7, 14, and 30 days of culture, and as a control which was used the culture medium of cells plated in flasks (2D). The values of protein reported in Figure 8 show for the control (CTRL) a typical phasic trend, while the samples, collected by the implants, report an increase in concentrations at 30 days of culture with a value higher than the relative control (Figure 6).

### 3.8. Vasculogenesis: Human-VEGF ELISA Test

**Figure 9:**
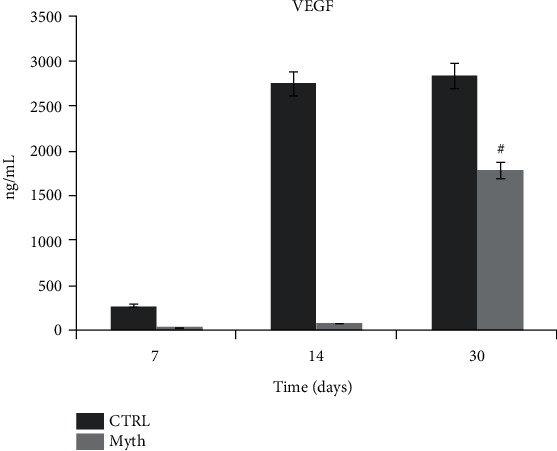
h-VEGF ELISA test of culture medium collected from 2D control (CTRL) and DPSC/Myth (Maipek Manufacturer Industrial Care, Naples, Italy) devices after 7, 14, and 30 days of cell culture. The concentration was expressed in pg/mL.

Vascular endothelial growth factor (VEGF) is a signal protein produced by cells that stimulate vasculogenesis and angiogenesis. The same protocol used for the h-OSTC ELISA test was performed for the evaluation of the concentration of VEGF released into the culture medium from DPSC/implant versus a control 2D. The values relative to Myth (Maipek Manufacturer Industrial Care, Naples, Italy) show an increasing trend during the time, with the highest peak at 30 days of culture, but the concentration is lower than that of the control for the respective times. The reason for that could be probably the search in the greater number of cells that the flask surface is able to contain with respect to implants (Figure 9).

## 4. Discussion

The bone-implant interface plays a critical role for good and lasting osteointegration. Many implant surfaces have been studied over the last decades. Among these, titanium alloy is the material most used because of its mechanical strength and its resistance to corrosion. In this research project, the capability of the Myth (Maipek Manufacturer Industrial Care, Naples, Italy) texture to induce the osteogenic process from DPSCs has been investigated; in particular, the fundamental aspects that regulate a full and long-term osseointegration at the bone-implant interface were examined.

The Myth (Maipek Manufacturer Industrial Care, Naples, Italy) implant results are completely biocompatible: they preserved the cell viability stimulating their proliferation. Immunofluorescence and SEM analyses allow a detailed view of cells onto implant surfaces and prove that implant texture enables cell adhesion and DPSC differentiation into osteoblastic morphology. After differentiation, DPSC growth on Myth (Maipek Manufacturer Industrial Care, Naples, Italy) surfaces implements extracellular matrix deposition and acts on the mineralization process, as the positivity for Alizarin Red staining revealed. The cell differentiation into the osteoblast and the activation of bone matrix formation were carried out in DPSCs seeded on Myth (Maipek Manufacturer Industrial Care, Naples, Italy) surfaces in an earlier stage with respect to the control. In particular, the key protein for bone tissue formation, the osteocalcin was already produced and released to be bound to ECM for mineralization. Also, the vasculogenesis process was carried out by cell-Myth (Maipek Manufacturer Industrial Care, Naples, Italy) devices, even if in a later stage with respect to the control [45–57].

DPSCs represent a suitable model for the study of bone differentiation thanks to their osteogenic capacity compared to other types of cells collected by the adult human body. This feature, together with their easy availability, high accessibility in the oral cavity, and resistance to cryopreservation, makes DPSCs very interesting for use in bone tissue engineering procedures in combination with scaffolds. Therefore, it could be of interest, after DPSC seeding on implants, to test their differentiation performance in a 3D culture system and to analyze their genetic behavior. The proliferation of osteoblasts around the implant is the basis of the osseointegration process. The sowing surface is decisive in guiding cellular activities, such as adhesion, diffusion, migration, and rearrangement: the cells acutely perceive the variability in the microenvironment and adapt to it. Differentiation and production of mineralized matrix involves the expression of a considerable number of genes, as well as the production of many different proteins that guide the process. Osteogenic differentiation is known to develop through spatiotemporal changes in the expression of the genes involved in this process. During its progression, specific markers reach one or more expression peaks depending on the maturation stage in which the cell is located. The expression of molecular markers associated with cell differentiation studied and monitored the synthesis and/or release of key molecules involved in this process and in the deposition of matrices by analyzing the expression of BAP, OPN, and OSTC. It was shown that the cells sown on implants had a significantly better expression of all three genes examined than in the control. This is probably due to the fact that the 3D cell culture simulates the physiological cell environment more accurately. In this study, stem cells are strongly stimulated to differentiate into osteoblasts, and this occurs in a few days (7 days); the latter is obtained thanks to the 3D cell culture, which is an excellent system for performing stem cell differentiation, because it significantly improves bone differentiation, improving the phenotypic expression of cells and the synthesis of mineralized matrix, and both the structure and composition of the implant, which promotes bone differentiation. As a result, the differentiation and deposition of the previous matrix led to a decrease in the OPN and OSTC gene expressions, which usually (without the aforementioned tools) decrease by day 21. Osteocalcin is one of the most abundant proteins in the bone. Angiogenesis is a crucial stage in ossification. Osteogenesis and angiogenesis are two processes that share different key regulators such as the vascular endothelial growth factor (VEGF). It has been highlighted how the level of this factor influences the time of cell growth suggesting a possible role in vasculogenesis. Most of the studies are aimed at assessing the rate of cell growth and not long-term biocompatibility, without considering that faster may not necessarily mean better [58–61]. Klos et al. [62] evaluated cell adhesion on laser-induced periodic surface structures. Human mesenchymal stem cells were grown on simple nanostructured surfaces. This process could appear slower on complex surfaces. The authors' study demonstrated how human mesenchymal stem cells were spatially controlled and how nanoscale structures influence surface wettability and protein adsorption. All these features could promote osteogenic differentiation. Di Carlo et al. [63] evaluated a titanium modified surface; their study focused on graphene oxide. The authors evaluated dental pulp stem cell viability, cytotoxicity, and osteogenic differentiation in the presence of graphene oxide-coated titanium surfaces. These surfaces demonstrated no significant differences with standard Ti disc surfaces [64–68]. The authors showed an increased secretion of PGE2 that could evidence a possible immunomodulatory role for graphene oxide. Diomede et al. [69] investigated the interaction between human periodontal stem cells and titanium surfaces using vascular endothelial growth factor and runt-related transcription factor 2. The authors in these cases demonstrated how the growth factor could influence and improve cell adhesion, osteogenic and angiogenic events, and osseointegration process. Sunarso et al. [70] evaluated the osteogenic capability of polyether-ether-ketone (PEEK). This study demonstrated that immobilization of phosphate or calcium increased the osteogenesis of rat mesenchymal stem cells compared with bare PEEK, including cell proliferation. Irastorza et al. [71] evaluated hDPSCs (human dental pulp stem cells), in combination with autologous plasma components, for in vitro bone generation on biomimetic titanium dental implant materials. The authors demonstrated how a combination of biomimetic rough titanium surfaces, with autologous plasma-derived fibrin-clot membranes such as PRF and/or insoluble PRGF formulations, improves osteoblastic cell differentiation, bone generation, anchorage, and osteointegration of titanium-made dental implants. Several modifications on the implant surfaces such as sandblasting, anodizing, acid attack, and calcium phosphate coverage have been designed in an attempt to improve the performance of the dental implant. Surface roughness is considered one of the most important characteristics for long-term implant stability. This study was conducted to test the osteoinductive potential of surfaces of dental implants on biological components.

## Figures and Tables

**Table 1:** Primers sequences for quantitative Real-Time Polymerase Chain Reaction (qRT-PCR).

Gene	Forward	Reverse	Ta
GAPDH	ggagtcaacggatttggtcg	cttcccgttctcagccttga	60°C
BAP	tcaaaccgagatacaagcac	ggccagacgaaagatagagt	56°C
COLL I	gaggctctgaaggtcccca	caccagcaataccaggagca	58°C
OPN	gccgaggtgatagtgtggtt	tgaggtgatgtcctcgtctg	58°C
BSP	ctggcacagggtatacagggttag	actggtgccgtttatgccttg	60°C
OSTC	ctcacactcctcgccctattg	cttggacacaaaggctgcac	60°C

## Data Availability

The data used to support the findings of this study are included within the article.
